# Clinical features of recurrent MOG antibody-associated cortical encephalitis in adults

**DOI:** 10.1007/s10072-024-07978-8

**Published:** 2025-01-22

**Authors:** Feiteng Qi, Guomin Xie, Yong Zhang

**Affiliations:** 1 Ningbo Medical Centre Lihuili Hospital, Ningbo, 315000 Zhejiang China; 2 The People’s Hospital of Xinchang, Shaoxing, 312500 Zhejiang China

**Keywords:** Adult, Cerebral cortical encephalitis, Myelin oligodendrocyte glycoprotein antibody

## Abstract

**Objective:**

To clarify the clinical features of recurrent myelin oligodendrocyte glycoprotein antibody-associated cortical encephalitis (MOGCE) in adults.

**Methods:**

We present an adult case of recurrent MOGCE and summarize the clinical symptoms, imaging findings, treatment and prognosis of this phenotype as per a systematic review of the literature.

**Results:**

We identified 9 adult patients with recurrent MOGCE. The mean age was 32 years, and 5/9 were male. Median time to recurrence was 6 months (range 2–36 months). The most common presentations of the first attack were headache (9/9), fever (8/9) and seizure (5/9). In most patients (6/9), presentations of recurrent attacks were different than those of the first attack. Relapses may affect more areas than the first attack, such as spine, brainstem, grey matter, and basal ganglia. All patients were seropositive for MOG antibodies during the first or second attack, with antibody titers ranging from 1:10 to 1:100. CSF white blood cell count and total protein were elevated in 6/9 patients. On MRI, 4/9 showed bilateral FLAIR hyperintense lesions, while 5/9 had unilateral lesions. Most patients demonstrated a positive response to treatment, and maintenance immunotherapy was added upon relapse.

**Conclusion:**

The clinical presentation of recurrent MOGCE is atypical, and most patients had different symptoms upon recurrence compared to the first episode. Factors contributing to the likelihood of disease recurrence remain unclear. Most recurrent MOGCE patients respond well to immunotherapy, and require long-term immunotherapy after recurrence.

Myelin oligodendrocyte glycoprotein antibody-associated disease (MOGAD) has been recognized in recent years as an inflammatory demyelinating disease of the central nervous system. MOGAD is an independent spectrum of disease distinct from multiple sclerosis and neuromyelitis optica spectrum disorder [1].The most common manifestations of MOGAD are optic neuritis, transverse myelitis, and acute disseminated encephalomyelitis; cortical encephalitis is rare. Ogawa et al. first reported a unique clinical type in 2017: benign unilateral cortical encephalitis, which presented with epileptic seizures [2]. Similar cases were subsequently reported and referred to as FLAIR-hyperintense lesions in anti-MOG-associated encephalitis with seizures (FLAMES) [3, 4].Fujimori et al. then found that some patients presented with bilateral cortical encephalitis (CE), expanding the spectrum of FLAMES [5].MOG antibody-associated cortical encephalitis (MOGCE) was proposed as a new spectrum, including both unilateral and bilateral CE [6]. In previously reported cases, most MOGCE patients responded well to high-dose glucocorticoids and recovered completely, and only a small percentage of patients relapsed [7, 8]. Recurrent MOGCE in adults is therefore uncommon, and its clinical features and prognosis have not been fully elucidated. Here, we report a case of recurrent MOGCE and systematically review the literature to better characterize this rare syndrome in adults.

## Case report

**Fig. 1:**
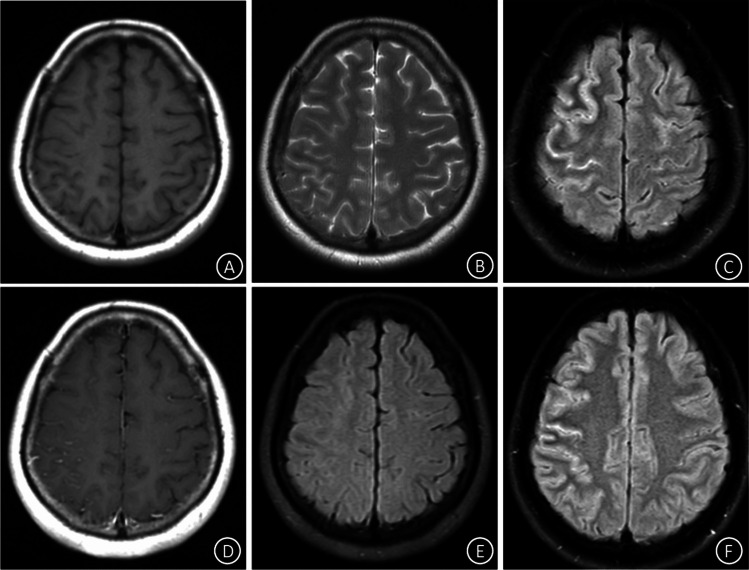
Cranial MRI findings. Upon first presentation, no obvious abnormal signal was seen in the (**A**) T1 or (**B**) T2 images. T2-FLAIR showed (**C**) hyperintensity in the right frontoparietal lobe and (**D**) spot enhancement. One month later, the high FLAIR signal had disappeared (**E**). Upon recurrence, the high FLAIR signal in right frontoparietal lobe reappeared (**F**)

A 46-year-old female was admitted to the hospital with sudden headache accompanied by numbness of the left upper limb. During this period, slurred speech with perioral numbness occurred, which resolved spontaneously in about 10 min. A nervous system physical examination showed the following: Clear mind, grade IV muscle strength in left upper limb with acupuncture pain reduction, normal muscle tension and tendon reflex in other limbs, negative Babinski and Chaddock signs, and negative stiff-neck signs. T1- and T2-weighted MRI showed no obvious abnormalities (Fig. 1A-B). T2-FLAIR imaging showed hyperintensity in the right frontoparietal lobe (Fig. 1C), as well as spot enhancement (Fig. 1D).

Lumbar puncture revealed an opening pressure of 200 mmH_2_O, 450 white blood cells (WBC)/µL, 111.1 mg/dL protein, and normal glucose levels in cerebrospinal fluid (CSF). Viral encephalitis was considered because the CSF bacterial culture was negative, and dexamethasone(10 mg/d for 7 days,then 5 mg/d for 7 days, intravenous infusion) and acyclovir (1.5g/d for 14 days, intravenous infusion) were given.

Lumbar puncture was repeated 12 days later, revealing pressure of 160 mmH2O, 20 WBC/µL, and protein 44.6 mg/dL. The patient was discharged with relief of headache. Cranial MRI was repeated one month later, and the high T2-FLAIR signal had basically disappeared (Fig. 1E).

After two months, the patient was again admitted to the hospital for headache. The headache was significantly aggravated compared with the previous one, and analgesic drugs were not effective. Lumber puncture revealed pressure of 150 mmH2O, 42 WBC/μL, and protein 39.3 mg/dL. Anti-MOG antibodies were positive in both serum (1:32) and CSF (1:3.2) by cell-based assay. Serum and CSF were negative for antibodies against Hu, Yo, Ri, N-methyl-D-aspartate receptor (NMDAR), glutamic acid decarboxylase, contactin-associated protein-like 2, leucine-rich glioma-inactivated 1-IgG, and aquaporin-4, as well as herpes virus. Cranial MRI revealed the reappearance of high T2-FLAIR signal in the right frontoparietal lobe (Fig. 1F). The patient was treated with dexamethasone(10 mg/d for 7 days,then 5 mg/d for 7 days, intravenous infusion) and mycophenolate mofetil(1.5 g/d, maintain throughout the follow-up period), which relieved her headache, and she reported no new complaints during the follow-up period.

## Methods

We searched PubMed for articles published from 2017 to 2023 containing ‘[cortical encephalitis] AND [MOG]’. Case inclusion criteria: (1) cortical T2-FLAIR hyperintensity, (2) positivity for MOG-IgG antibodies in serum and/or CSF as assessed by cell-based assay, (3) older than 16 years. Exclusion criteria: (1) other infectious/autoimmune cases, (2) available data were not adequately reported in the publication. Authors' disagreements regarding the inclusion of cases were resolved through discussion. A total of 9 cases were included, including the case described above. Clinical information for the first attack are shown in Table 1 and for recurrence in Table 2.

**Table 1:** Clinical features of first attack

Case	Age/ Sex	Clinical symptoms of first attack				Laboratory tests				Imaging			Treatment
		Fever	Headache	seizure	Other symptoms	CSF cells	CSF protein	Serum MOG	Other abs	region	Unilateral/ bilateral	Enhancement	
Case1 [27]	20/M	+	+	+	coma	80	0.48	NA	NA	frontotemporal parietal	bilateral	NA	dexamethasone
Case2 [28]	39/M	+	+	+	-	75	0.69	NA	NA	frontal, temporal, parietal	bilateral	+	IVIG
Case3 [29]	55/M	+	+	+	Aphasia, Hemiparesis, hypoesthesia	2	0.48	NA	-	temporal, parietal, occipital	Unilateral	-	IVIG + HIMP
Case4 [9]	18/M	+	+	-	vision loss, palsy of two legs	0	0.19	1:10	-	Frontal, parietal	bilateral	+	HIMP + IVIG + PE;
Case5 [9]	16/F	+	+	-	vision loss	18	0.31	1:32	-	right parietal, temporal, occipital	Unilateral	-	HIMP
Case6 [24]	31/F	+	+	+	nausea, vomiting	465	0.92	1:10	-	left temporal, parietal, and occipital	Unilateral	-	HIMP
Case7 [10]	29/M	+	+	+	-	112	0.45	1:100 In CSF	NMDAR:1:1 GFAP:1:32	Frontal	bilateral	NA	HIMP
Case8 [30]	34/F	+	+	-	memory loss, and left upper extremity weakness	113	0.63	+	NA	right temporal lobe	Unilateral	+	HIMP
Case9	46/F	-	+	-	numbness and weakness of the left upper limb	450	1.11	NA	NA	Right frontal, parietal	Unilateral	+	dexamethasone

*M*, male; *F*, female; *CSF*, cerebrospinal fluid; *MOG*, myelin oligodendrocyte glycoprotein; *abs*, antibodies; *NMDAR*, N-methyl-D-aspartate receptor; *GFAP*, glial fibrillary acidic protein; *HIMP*, high-dose intravenous methylprednisolone; *IVIG*, intravenous immunoglobulins; *NA*, not available; *MMF*, mycophenolate mofetil.

**Table 2:** Clinical features of second attack

Case	Relapse(m)	Clinical symptoms of relapse				Laboratory tests				Imaging			Treatment
		Fever	Headache	seizure	Other symptoms	CSF cells	CSF protein	Serum MOG	Other abs	region	Unilateral/ bilateral	Enhancement	
Case1 [27]	36	-	-	-	Dizziness,unsteady gait	4	0.51	1:10	-	brainstem	Unilateral	NA	HIMP + MMF
Case2 [28]	2	-	-	-	vision impaired	-	0.53	1:10	-	Parietal,cerebellar dentate nucleus,optic nerve	bilateral	+	HIMP
Case3 [29]	4	-	-	-	dizziness	16	NA	1:20	-	temporal	Unilateral	NA	HIMP
Case4 [9]	19	+	+	-	vision loss, palsy of two legs	NA	NA	1:32	-	Frontal, parietal	bilateral	-	PE + Rituximab
Case5 [9]	7	+	+	-	vision loss	NA	NA	1:100	-	right parietal, temporal, occipital	Unilateral	-	MMF
Case6 [24]	2	-	-	-	paresthesias, blurry vision, ataxia	NA	NA	1:10	-	thalamus, basal ganglia, centrum semiovale	bilateral	NA	MMF
Case7 [10]	10	+	+	+	-	NA	NA	1:10	NMDAR:1:1 GFAP:1:32	Frontal	bilateral	NA	HIMP + MMF
Case8 [30]	6	-	-	-	transverse myelitis	-	-	NA	NA	thoracic spinal cord	-	-	HIMP + plasma exchange + tacrolimus
Case9	2	-	+	-	-	42	0.39	1:32	-	Right frontal, parietal	Unilateral	NA	Dexamethasone + MMF

*CSF*, cerebrospinal fluid; *MOG*, myelin oligodendrocyte glycoprotein; *abs*, antibodies; *NMDAR*, N-methyl-D-aspartate receptor; *GFAP*, glial fibrillary acidic protein; *HIMP*, high-dose intravenous methylprednisolone; *IVIG*, intravenous immunoglobulins; *NA*, not available; *MMF*, mycophenolate mofetil.

## Results

### Demographics and clinical features

Of the nine adult patients identified with recurrent MOGCE, the mean age was 32 years (range 16–55 years) and 5/9 (55.6%) were male. The median time to recurrence was 6 months (range 2–36 months). The most common presentations of the first attack were headache (9/9), fever (8/9), and seizure (5/9). Other symptoms included hemiparesis(4/9), visual disturbances(2/9), numbness of the limbs(2/9), and aphasia(1/9). Most patients had different symptoms upon recurrence compared to the first episode (6/9). Five cases initially presented with MOGCE and experienced MOGCE again at recurrence. Although both presented with CE, the clinical manifestations were not the same. In subsequent episodes, there was a higher incidence of neurological deficits, such as visual impairment(3/9), and a lower incidence of headache (4/9) and fever (3/9).

### Laboratory characteristics and imaging

All patients were positive for MOG antibodies in the serum during the first or second attack, with antibody titers ranging from 1:10 to 1:100. Antibody titers were not found to be associated with disease severity or treatment response. In five patients, MOG antibodies were not tested at the time of the first attack and were found to be positive at the time of relapse. One patient was also positive for NMDAR and glial fibrillary acidic protein antibodies.

CSF WBC count was elevated (> 8 cells/μL) in 7/9 patients, with a median of 80 cells/μL (range 0–465 cells/μL). CSF total protein was elevated (> 0.4 g/L) in 7/9 patients, with a median of 0.48 g/L (range 0.19–1.11 g/L).

On brain MRI, 4/9 showed bilateral FLAIR hyperintense lesions, while 5/9 had unilateral lesions. Relapses may affect more areas than the first attack, such as spine, brainstem, grey matter, and basal ganglia. Not all lesions had obvious contrast enhancement on enhanced MRI.

### Treatment and outcomes

Patients were treated with short-term first-line immunotherapy (high-dose intravenous methylprednisolone, dexamethasone, intravenous immunoglobulins, or plasma exchange) after the first attack. Most cases demonstrated a positive response to treatment. Following relapse, tacrolimus, mycophenolate mofetil, and rituximab were added to the treatment regimen, along with steroids. Only one patient experienced moderate paralysis due to spinal cord involvement at the time of relapse; the others recovered well.

## Discussion

This study is the first to describe a cohort of recurrent MOGCE cases. We discuss clinical presentations, laboratory test results, and imaging features, and compare initial and recurrent episodes. This study improves our understanding of this unique MOGAD phenotype and will help clinicians identify MOGCE in practice. MOGCE still presents some challenges, including misdiagnosis, identification of recurrence factors, and necessity of long-term immunotherapy.

The clinical manifestations of MOGCE are not well-defined, and the initial diagnosis in many cases reported in the literature was delayed or incorrect. Headache is the most common presentation; 100% of MOGCE patients reviewed in this study had headache at first attack, and 44.4% had headache at relapse. MOGCE typically involves severe headache, progressive disease course, and rapid alleviation with immunotherapy [9]. Fever is the second most common clinical symptom, which contributes to the common misdiagnosis of infectious encephalitis. Yuqing et al. reported that 66.7% of MOGCE patients presented with fever [10], which is lower than the 88.9% observed in the present study. It is possible that patients with fever are more likely to relapse; however, additional studies with larger sample sizes are needed to confirm this.The third most common clinical presentation was seizures. They are typically focal-onset, most often present as motor seizures, ictal aphasia, or somatosensory symptoms [11], and have been reported to progress to bilateral tonic–clonic seizures in as many as 77% of MOGCE patients [3, 12].Although the most common clinical symptoms at the time of the first attack were headache, fever, and seizure, these were not the predominant symptoms at the time of recurrence. At relapse, symptoms were more varied and included neurological symptoms such as visual impairment and limb paralysis.

MOG antibody positivity is key to the diagnosis of MOGCE. Valencia-Sanchez et al. reported that initial misdiagnosis occurred in 47% of cases, as isolated CE did not always prompt consideration of central nervous system demyelination and MOG-IgG testing [11].Of the nine patients included in our study, five were initially misdiagnosed because they were not tested for MOG antibodies at the time of the first attack; MOGCE diagnosis was confirmed only at the time of relapse. It has been reported that serum MOG-IgG titer is correlated with disease activity, and that titer is higher in the acute stage than in the remission stage [13]. A high antibody titer [14] and persistent antibody positivity [15] have been proposed to be correlated with the likelihood of recurrence. However, the present cohort of patients with recurrent MOGCE did not display unusually high MOG titers (range 1:10 to 1:100). Additional, multi-center, large-sample studies will be needed to further explore the relationship between MOG antibody titer and likelihood of recurrence.

MOGCE often presents with clinical meningoencephalitis symptoms and abnormal CSF findings mimicking central nervous system infection [16]. Elevated CSF WBC count is common in MOGCE, but rare in other cerebral demyelinating diseases [17]. It has been reported that CSF WBC count increased in 92% of MOGCE cases [18]. In the present review, CSF WBC count was found to be elevated in 7/9 patients (77.8%). CSF inflammation is easily confused with infectious diseases of the nervous system, making early diagnosis difficult. Adequate etiological examination is needed to exclude infection [19]. There was insufficient data to conclude whether CSF WBC count is associated with recurrence; 4/9 cases did not have this information available.

Lesions in MOGCE are typically distributed in the peripheral territories of the anterior cerebral arteries (bilateral) and the peripheral territories of the middle cerebral arteries (unilateral) [20, 21].In the present study, four presenting with bilateral cortical lesions and five with unilateral cortical lesions at the first attack. At relapse, four patients presented with lesions in spinal, brainstem, grey matter, and basal ganglia regions and the other five patients presented with cortical lesions. Similarly, Horita et al. reported that MOGCE can manifest with a variety of phenotypes over time in the same patient [22]. An Austrian case report also showed extensive, predominantly cortical demyelinating lesions with extension into subcortical white matter [23].Together these observations demonstrate that MOGCE is a disease that can affect multiple locations within the central nervous system over time, and is not limited to the cortex.

Treatment responses vary widely in MOGCE. Intravenous high-dose methylprednisolone and immunoglobulins comprise the standard first-line treatment to combat MOGCE in the acute phase, and usually result in significant improvements in clinical symptoms and imaging. In some patients, however, recurrence occurred after withdrawal from therapy, indicating a high state of disease activity [24].In our study, none of the patients with recurrent MOGCE were taking long-term immunosuppressive drugs after their first onset. Currently, there is no consensus on the necessity of long-term maintenance therapy for MOGCE patients at first attack [25, 26]. When recurred, 8 out of 9 patients had a good prognosis after immunotherapy in our study. Therefore, considering the low recurrence rate of MOGCE and the obvious effect of immunotherapy after recurrence, patients with MOGCE may not need long-term immune maintenance therapy at the first episode.But this conclusion needs further confirmation. It is clear that recurrent MOGCE requires long-term use of steroids or immunosuppressive therapy, such as mycophenolate mofetil, rituximab, and tacrolimus, to prevent relapse again.

This study has some limitations. First, it is a retrospective literature review in which no comparison was made between relapsed and non-relapsed MOGCE. Second, the sample size was relatively small. Finally, it was unable to identify a factor that can predict whether or not a patient with initial MOGCE will experience relapse. Further prospective studies with larger sample sizes and longer follow up are warranted to estimate the likelihood of MOGCE relapse.

In conclusion, recurrent MOGCE is a disease that can affect multiple locations within the central nervous system over time, and is not limited to the cortex. Factors contributing to the likelihood of disease recurrence remain unclear. Patients with recurrent MOGCE do not require unusually high MOG titers. Whether long-term treatment is needed during the first episode is uncertain, but long-term immunotherapy is required after recurrence. Currently, research on recurrent MOGCE is limited and requires further exploration.
